# A modified viscosity implicit-type proximal point algorithm for monotone inclusions and asymptotically nonexpansive mappings in Hadamard spaces

**DOI:** 10.1186/s13660-018-1825-5

**Published:** 2018-09-12

**Authors:** Shih-sen Chang, Jen-Chih Yao, Ching-Feng Wen, Lin Wang

**Affiliations:** 1 Center for General Education, China Medical University, Taichung, Taiwan; 2 Center for Fundamental Science; and Research Center for Nonlinear Analysis and Optimization, Kaohsiung Medical University, Kaohsiung, Taiwan; 3 Department of Medical Research, Kaohsiung Medical University Hospital, Kaohsiung, Taiwan; 4 College of Statistics and Mathematics, Yunnan University of Finance and Economics, Kunming, China

**Keywords:** 47H09, 47J25, 47H05, 47J05, 47J20, 65K05, Hadamard space, CAT(0) space, Viscosity implicit approximation methods, Monotone mapping, Proximal point algorithm

## Abstract

The purpose of this article is to propose a modified viscosity implicit-type proximal point algorithm for approximating a common solution of a monotone inclusion problem and a fixed point problem for an asymptotically nonexpansive mapping in Hadamard spaces. Under suitable conditions, some strong convergence theorems of the proposed algorithms to such a common solution are proved. Our results extend and complement some recent results in this direction.

## Introduction

One of the most important parts in nonlinear and convex analysis is monotone operator theory. The following zero problem is one of the most important problems in monotone operator theory:

1.1 $$ \text{to find } x \in D(A)\quad \text{such that } 0 \in A(x), $$

where *A* is a monotone operator. This problem includes, as special cases, convex programming, variational inequalities, split feasibility problem and minimization problem. To be more precise, some concrete problems in machine learning, image processing and linear inverse problem can be modelled mathematically as this form [1, 2].

A popular method for approximation of a zero of a monotone operator *A* is the proximal point algorithm, which was introduced in Hilbert space *H* by Martinet [1] and Rockafellar [2] as follows:

1.2 $$ x_{n+1} - x_{n} \in\lambda_{n} A(x_{n}), \quad x_{0} \in H, $$

where $\{\lambda_{n}\}$ is a sequence of positive real numbers. Rockafellar [2] (see, also Bruck and Reich [3]) proved that the sequence generated by the proximal point algorithm is weakly convergent to a zero of the monotone operator *A* provided $\lambda_{n} \ge\lambda> 0$, for each $n \ge1$. Güler’s counterexample [4] (see, also Bauschke [5]) showed that the sequence generated by (1.2) does not necessarily converge strongly even if the maximal monotone operator is the subdifferential of a convex, proper, and lower semicontinuous function.

Another algorithm for approximation a zero of the monotone operator *A* is the viscosity approximation method which was proposed by Takahashi [6] in Banach spaces

1.3 $$ x_{n+1} = \alpha_{n} f(x_{n}) + (1 - \alpha_{n})J^{A}_{\lambda_{n}} x_{n}, $$

where *f* is a contractive mapping and $J^{A}_{\lambda}= (I + \lambda A)^{-1}$, $\lambda> 0$ is the resolvent of *A*. Under suitable conditions, he proved strong convergence of (1.3) in Banach spaces.

In 2006, Maingé [7] also studied the following more general iteration:

1.4 $$ x_{n+1} = \alpha_{n} T_{n} (x_{n}) + (1 - \alpha_{n})J^{A}_{\lambda_{n}} x_{n}, $$

where $\{T_{n}\}$ is a family of nonexpansive mappings. Under suitable conditions he proved strong convergence of (1.4) in Banach spaces.

In 2013, Bačák [8] proved the △-convergence of proximal point algorithm in CAT(0) spaces when the operator *A* is the subdifferential of a convex, proper, and lower semicontinuous function. Recently, some more general modified proximal point algorithms in CAT(0) have been considered by Li et al. [9], Cholamjiak [10], Chang et al. [11–13], Khatibzadeh et al. [14–16], Cholamjiak et al. [17], Suparatulatorn et al. [18] and Bačak and Reich [19], Sahu [20].

Very recently, Ranjbar and Khatibzadeh [16] introduced the concept of monotone operator in Hadamard space *X*, and considered some properties of a monotone operator and its resolvent operator, then proposed the following proximal point algorithm:

1.5 $$ \left \{ \textstyle\begin{array}{l} x_{n+1} = J_{\lambda_{n}} x_{n},\\ x_{0} \in X, \end{array}\displaystyle \right . $$

where $A : X \to2^{X^{*}}$ is a multi-valued monotone operator, $\{ \lambda_{n}\}$ is a sequence of positive real numbers and $J_{\lambda}$ is the resolvent of *A* defined by (2.8) (see, Sect. 2). Under suitable conditions they proved that the sequence $\{x_{n}\}$ △-converges or converges strongly to a zero of *A*.

Motivated and inspired by the research going on in this direction, the purpose of this article is to propose a modified viscosity implicit-type proximal point algorithm for approximating a common solution of monotone inclusion problem and fixed point problem for an asymptotically nonexpansive mapping in Hadamard space which is also a unique solution of some variational inequality problems. Our results extend and complement the main results of Bačák [8], Khatibzadeh et al. [14–16].

## Preliminaries and Hadamard spaces

Let $(X, d)$ be a metric space and $x, y \in X$. A geodesic path joining *x* to *y* is an isometry $c : [0, d(x, y)] \to X$ such that $c(0) = x$ and $c(d(x, y)) = y$. The image of a geodesic path joining *x* to *y* is called a geodesic segment between *x* and *y*. The metric space $(X, d)$ is said to be a geodesic space, if every two points of *X* are joined by a geodesic. *X* is said to be uniquely geodesic space, if there is exactly one geodesic joining *x* and *y* for each $x, y \in X$.

A geodesic space $(X, d)$ is a CAT(0) space, if and only if the following “CN-inequality” holds:

2.1 $$ d^{2}\bigl((1-t)x \oplus t y, z\bigr)\le(1-t) d^{2}(x, z) + t d^{2}(y,z) - t (1-t) d^{2}(x, y) $$

for all $x, y,z \in X$ and all $t \in[0, 1]$ [21].

It is well known that any complete and simply connected Riemannian manifold having nonpositive sectional curvature is a CAT(0) space. The Hilbert ball with the hyperbolic metric is an important example of a CAT(0) space [22]. Other examples of CAT(0) spaces include pre-Hilbert spaces, *R*-trees, Euclidean buildings [23].

A complete CAT(0) space is often called an *Hadamard space*. We write $(1-t)x \oplus ty$ for the unique point *z* in the geodesic segment joining from *x* to *y* such that $d(x, z) = td(x, y)$ and $d(y, z) = (1-t)d(x, y)$. We also denote by $[x, y]$ the geodesic segment joining *x* to *y*, that is, $[x, y] = \{(1-t) x \oplus ty: 0 \le t \le 1 \}$. A subset *C* of a CAT(0) space is convex if $[x, y] \subset C$ for all $x, y \in C$.

Berg and Nikolaev [24] introduced the following concept of quasilinearization in CAT(0) space *X*.

- Denote a pair $(a,b)\in X\times X$ by $\overrightarrow {ab}$ and call it a *vector*.
- *Quasi-linearization* in CAT(0) space *X* is defined as a mapping $\langle\cdot,\cdot\rangle: (X\times X)\times(X\times X)\to\mathbf {R}$ such that
  2.2 $$ \langle\overrightarrow{ab},\overrightarrow{cd}\rangle=\frac {1}{2} \bigl(d^{2}(a,d)+d^{2}(b,c)-d^{2}(a,c)-d^{2}(b,d) \bigr) $$
  for all $a,b,c,d\in X$.
  It can easily be verified that $\langle\overrightarrow{ab}, \overrightarrow{ab} \rangle= d^{2}(a,b)$, $\langle\overrightarrow{ba}, \overrightarrow{cd} \rangle= - \langle\overrightarrow{ab}, \overrightarrow{cd} \rangle$ and $\langle\overrightarrow{ab}, \overrightarrow{cd} \rangle= \langle\overrightarrow{ae}, \overrightarrow{cd} \rangle+ \langle\overrightarrow{eb}, \overrightarrow{cd} \rangle$ for all $a, b, c, d, e \in X$.
- We say that *X* satisfies the Cauchy–Schwarz inequality if
  2.3 $$ \langle\overrightarrow{ab}, \overrightarrow{cd} \rangle\le d(a, b) d(c, d), \quad\forall a, b, c, d \in X. $$
  It is well known [24, Corollary 3] that a geodesically connected metric space is a CAT(0) space if and only if it satisfies the Cauchy–Schwarz inequality.

The following inequalities can be proved easily.

### Lemma 2.1

*Let* *X* *be a* $\operatorname{CAT}(0)$ *space*. *For all* $x, y, z\in X$ *and* $t, s \in [0,1]$, *we have the following*:

1. $d(t x\oplus(1-t)y, z)\leq t d(x,z) +(1-t) d(y,z)$,
2. $d(t x\oplus(1-t)y, s x\oplus(1-s)y)= |t -s| d(x,y)$,
3. $d(t x\oplus(1- t)y, t u\oplus(1- t)w)\leq t d(x, u) +(1- t) d(y, w)$,
4. *by using equality* (2.2), *inequality* (2.1) *can be written as*
  2.4 $$ \begin{aligned}[b] d^{2}\bigl((1-t)x \oplus t y, z\bigr) & \le(1-t) d^{2}(x, z) + t d^{2}(y,z) - t (1-t) d^{2}(x, y) \\ & = (1-t)^{2} d^{2}(x, z) + t^{2} d^{2}(y,z) + 2t (1-t)\langle\overrightarrow {xz}, \overrightarrow{yz} \rangle. \end{aligned} $$

- By using quasilinearization, Kakavandi and Amini [25] introduced the concept of dual space of a Hadamard space *X* as follows.
  Consider the mapping $\Theta: {\mathbf{R}} \times X \times X \to C(X, {\mathbf{R}})$ defined by
  $$\Theta(t, a, b) (x) = t \langle\overrightarrow{a b}, \overrightarrow{a x} \rangle\quad (t \in\mathbf{R}, a, b, x \in X), $$
  where $C(X, \textbf{R})$ is the space of all continuous real-valued functions on *X*. Then the Cauchy–Schwarz inequality implies that $\Theta(t, a, b)$ is a Lipschitz function with Lipschitz semi-norm $L(\Theta(t, a, b)) = |t|d(a, b)$ ($t \in\textbf {R}$, $a, b \in X$), where
  $$L(\phi) = \sup \biggl\{ \frac{\phi(x) - \phi(y)}{d(x,y)} : x, y \in X, x \neq y\biggr\} $$
  is the Lipschitz semi-norm for any function $\phi: X \to\textbf{R}$. A pseudometric *D* on $\textbf{R} \times X \times X$ is defined by
  $$D\bigl((t, a, b), (s, c, d)\bigr) = L\bigl(\Theta(t, a, b) - \Theta(s, c, d) \bigr)\quad (t, s \in\textbf{R}, a, b, c, d \in X). $$
  For an Hadamard space $(X, d)$, the pseudometric space $( \textbf{R} \times X \times X,D)$ can be considered as a subspace of the pseudometric space of all real-valued Lipschitz functions $(\operatorname{Lip}(X, \textbf{R}), L)$. By [25, Lemma 2.1], $D((t, a, b), (s, c, d)) = 0$ if and only if $t \langle\overrightarrow{a b}, \overrightarrow{x y}\rangle= s \langle\overrightarrow{c d}, \overrightarrow{x y}\rangle$, $\forall x, y \in X$ Thus, *D* induces an equivalence relation on $\textbf{R} \times X \times X$, where the equivalence class of $(t, a, b)$ is
  $$[t\overrightarrow{ a b}] =\bigl\{ s\overrightarrow{cd}: D\bigl((t,a,b), (s,c,d) \bigr) = 0\bigr\} . $$
  The set $X^{*} = \{[t\overrightarrow{ab}]: (t, a, b) \in\textbf{R} \times X \times X\}$ is a metric space with metric
  $$D\bigl([t\overrightarrow{ab}], [s\overrightarrow{cd}]\bigr) : = D\bigl((tab), (scd)\bigr), $$
  which is called the dual space of $(X, d)$. It is clear that $[\overrightarrow{aa}] = [\overrightarrow{bb}]$ for all $a, b \in X$. Fix $x \in X$, we write $0 = [\overrightarrow{xx}]$ as the zero of the dual space.

### Example

In [25], it is shown that the dual of a closed and convex subset of a Hilbert space *H* with nonempty interior is *H* and $t(b - a)\equiv[t \overrightarrow{ab}]$ for all $t \in\textbf{R}$, $a,b \in H$.

Note that $X^{*}$ acts on $X \times X$ by

$$\bigl\langle x^{*}, \overrightarrow{xy} \bigr\rangle = t \langle\overrightarrow {ab}, \overrightarrow{xy}\rangle\quad \bigl(x^{*} = [t\overrightarrow{ab}]\in X^{*}, x, y \in X\bigr). $$

Also, we use the following notation:

$$\bigl\langle \alpha x^{*} + \beta y^{*}, \overrightarrow{xy} \bigr\rangle = \alpha \bigl\langle x^{*}, \overrightarrow{xy} \bigr\rangle + \beta\bigl\langle y^{*},, \overrightarrow{xy} \bigr\rangle \quad \bigl(\alpha, \beta\in \textbf{R}, x, y \in X, x^{*}, y^{*} \in X^{*}\bigr). $$

Let $\{x_{n}\}$ be a bounded sequence in a Hadamard space *X*. For $x \in X$, define $r(x, \{x_{n}\}) := \limsup_{n \to\infty}d(x, x_{n})$. The asymptotic radius $r(\{x_{n}\})$ of $\{x_{n}\}$ is defined by $r(\{x_{n}\} ) = \inf\{r(x, \{x_{n}\}) : x \in X\}$, and the asymptotic center $A(\{x_{n}\})$ of $\{x_{n}\}$ is the set $A(\{x_{n}\}) = \{x \in X : r(x, \{x_{n}\})\} = r(\{x_{n}\})$. It is well known that in a CAT(0) space, $A(\{x_{n}\})$ consists of exactly one point (see [26, Proposition 7]). A sequence $\{x_{n}\}$ in *X* is said to be △-convergent to a point *w*, if *w* is the unique asymptotic center of every subsequence $\{u_{n}\}$ of $\{x_{n}\}$. This is written as $\triangle\text{-}\lim_{n \to\infty} x_{n} = w$. (We denote it by $\{ x_{n}\} \rightharpoonup w$).

### Lemma 2.2

*Let* *X* *be an Hadamard space*. *The following statements hold*.

1. [27] *Every bounded sequence in a Hadamard space always has a* Δ-*convergent subsequence*.
2. [28] *A sequence* $\{x_{n}\} \bigtriangleup$-*converges to* $x \in X$ *if and only if*
  $$\limsup_{n \to\infty}\langle\overrightarrow{xx_{n}}, \overrightarrow {xy}\rangle\le0,\quad \forall y \in X. $$

- Let *C* be a nonempty closed convex subset of an Hadamard space *X*. The *metric projection* $P_{C}: X\to C$ is defined by
  2.5 $$ u=P_{C}(x)\quad \Longleftrightarrow\quad d(u,x)=\inf\bigl\{ d(y,x):y\in C\bigr\} ,\quad x\in X. $$

### Lemma 2.3

([24])

*Let* *C* *be a nonempty closed and convex subset of an Hadamard space* *X*, $x\in X$ *and* $u\in C$. *Then* $u=P_{C}(x)$ *if and only if*

2.6 $$ \langle\overrightarrow{yu},\overrightarrow{ux}\rangle\geq0, \quad \forall y\in C. $$

### Definition 2.4

Let *X* be an Hadamard space with dual $X^{*}$ and $A: X \to2^{X^{*}}$ be a multi-valued mapping with domain $D(A) : = \{x \in X : A(x) \neq\emptyset\}$.

1. *A* is said to be monotone [15], if for all $x, y \in D(A)$, $x^{*} \in Ax$ and $y^{*} \in Ay$
  2.7 $$ \bigl\langle x^{*} - y^{*}, \overrightarrow{yx} \bigr\rangle \ge0. $$
  The multi-valued monotone operator $A : X \to2^{X^{*}}$ is maximal if there exists no monotone operator $B : X \to2^{X^{*}}$ such that $\operatorname{graph}(B)$ properly contains $\operatorname{graph}(A)$.
2. Let $\lambda> 0$ and $A : X \to2^{X^{*}}$ be a set-valued operator. The resolvent of *A* of order *λ* is the set-valued mapping $J_{\lambda}: X \to2^{X}$ defined by
  2.8 $$ J_{\lambda}(x) := \biggl\{ z \in X: \biggl[\frac{1}{\lambda} \overrightarrow{zx}\biggr] \in Az\biggr\} . $$
3. *A* is said to satisfy the range condition [15] if, for each $\lambda> 0$ $D(J_{\lambda}) =X$, where $J_{\lambda}$ is the resolvent of *A* defined by (2.8).

### Remark

It has shown in [9] if *A* is a maximal monotone operator on an Hadamard space, then *A* satisfies the range condition.

### Definition 2.5

Let $T: X \to X$ be a mapping. *T* is said to be:

1. nonexpansive if
  2.9 $$ d(Tx, Ty) \le d(x,y), \quad\forall x, y \in X; $$
2. firmly nonexpansive if
  2.10 $$ d^{2}(Tx,Ty) \le\langle\overrightarrow{Tx Ty}, \overrightarrow {xy} \rangle,\quad\forall x, y \in X; $$
3. asymptotically nonexpansive, if there is a sequence $\{k_{n}\} \subset[1, \infty)$ with $k_{n} \to1$ as $n \to\infty$ such that
  2.11 $$ d\bigl(T^{n} x, T^{n} y\bigr) \le k_{n} d(x, y), \quad\forall n \ge1, x, y \in X. $$

### Lemma 2.6

1. (*By the definition of firmly nonexpansive mapping and Cauchy–Schwarz inequality*, *it is clear that*) *each firmly nonexpansive mapping* *T* *is nonexpansive*.
2. [29] *Let* *C* *be a closed convex subset of a Hadamard space* *X* *and* $T : C \to X$ *be an asymptotically nonexpansive mapping*. *Let* $\{x_{n}\}$ *be a bounded sequence in* *C* *such that* $x_{n} \rightharpoonup p$ *and* $\lim_{n \to\infty} d(x_{n}, Tx_{n}) = 0$, *then* $Tp = p$.

### Theorem 2.7

([15])

*Let X be an Hadamard space and* $J_{\lambda}$ *be the resolvent of the operator* *A* *of order* *λ*. *Then*

(i) *for any* $\lambda> 0$, $\mathrm{R}(J_{\lambda}) \subset D(A)$, $\operatorname{Fix}(J_{\lambda}) = A^{-1}(0)$, *where* $\mathrm{R}(J_{\lambda})$ *is the range of the mapping* $J_{\lambda}$ *and* $\operatorname{Fix}(J_{\lambda})$ *is the set of fixed points of* $J_{\lambda}$;
(ii) *if* *A* *is monotone*, *then*, *for each* $\lambda> 0$, $J_{\lambda}$ *is a single*-*valued and firmly nonexpansive mapping*.

### Remark 2.8

It is well known that if *C* is a nonempty and closed convex subset of a CAT(0) space and $T: C \to C$ is a nonexpansive mapping, then $\operatorname{Fix}(T)$ is closed and convex. Thus, if *A* is a monotone operator on a CAT(0) space *X*, then, by the conclusions (i) and (ii) of Theorem 2.7, $A^{-1}(0)$ is closed and convex.

### Lemma 2.9

([30])

*Let* $\{s_{n}\}$ *be a sequence of nonnegative real numbers*, $\{\alpha_{n}\}$ *and* $\{\beta_{n}\}$ *be sequences of real numbers in* $(0, 1)$ *with* $\sum_{n=1}^{\infty}\alpha_{n} = \infty$, *and* $\{t_{n}\}$ *be a sequence of real numbers such that*

$$s_{n+1} \le(1-\alpha_{n})s_{n} + \alpha_{n} t_{n} + \beta_{n},\quad \forall n \ge1. $$

*If* $\limsup_{n \to\infty} t_{n} \le0$, *and* $\sum_{n =1}^{\infty}\beta _{n} < \infty$, *then* $\lim_{n\to\infty} s_{n} = 0$.

## The main results

Now, we are in a position to give the main results in this paper.

### Theorem 3.1

*Let* *X* *be an Hadamard space with dual* $X^{*}$. *Let* $T: X \to X$ *be an asymptotically nonexpansive mapping with sequence* $\{k_{n}\} \subset[1, \infty)$ *and* $\lim_{n \to\infty} k_{n} =1$, $A : X \to2^{X^{*}}$ *be a multi*-*valued monotone operator satisfying the range condition and* $f: X \to X$ *be a contractive mapping with contractive coefficient* $\gamma\in(0, 1)$ *and*, *for arbitrary initial point* $x_{1} \in X$, *let* $\{x_{n}\}$ *be the sequence generated by*

3.1 $$ \left \{ \textstyle\begin{array}{l} y_{n} = J_{\lambda}(x_{n}),\\ x_{n+1} = \alpha_{n} f(J_{\lambda}(x_{n})) \oplus(1- \alpha_{n})T^{n}(\beta_{n} J_{\lambda}(x_{n}) \oplus(1- \beta_{n}) J_{\lambda}(x_{n+1})), \end{array}\displaystyle \right .\quad\forall n \ge1, $$

*where* $\{\alpha_{n}\}$ *and* $\{\beta_{n}\}$ *are real sequences in* (0, 1) *satisfying the following conditions*:

(i) $\lim_{n \to\infty} \alpha_{n} = 0$;
(ii) $\sum_{n = 1}^{\infty}\alpha_{n} = \infty$;
(iii) $\lim_{n\to\infty} \frac{k_{n} - 1}{\alpha_{n}} = 0$;
(iv) $\frac{|\alpha_{n} -\alpha_{n -1}|}{\alpha_{n} ^{2}} \to0$, *as* $n \to\infty$;
(v) *T* *is uniformly asymptotically regular i*.*e*., *for any* $x \in X$
  $$\lim_{n \to\infty} d\bigl(T^{n} x, T^{n+1}x\bigr) = 0. $$

*If* $\Gamma: = \operatorname{Fix}(T)\cap A^{-1}(0) \neq\emptyset$, *then* $\{x_{n}\}$ *converges strongly to* $x^{*} \in\Gamma$ *which solves the following variational inequality*:

3.2 $$ \bigl\langle \overrightarrow{x^{*}f\bigl(x^{*}\bigr)}, \overrightarrow{q x^{*}}\bigr\rangle \ge0, \quad \forall q\in\Gamma. $$

### Proof

(I) *First we prove that* $\{x_{n}\}$ *defined by* (3.1) *is well defined.*

In fact, for each $n \ge1$, let us define a mapping $T_{n}: X \to X$ by

$$T_{n} (x)= \alpha_{n} f\bigl(J_{\lambda}(x_{n}) \bigr) \oplus(1- \alpha_{n})T^{n}\bigl(\beta_{n} J_{\lambda}(x_{n}) \oplus(1- \beta_{n})J_{\lambda}(x) \bigr). $$

Since *T* is asymptotically nonexpansive and $J_{\lambda}$ is nonexpansive, we have

$$\begin{aligned} d(T_{n} x , T_{n} y) &= d\bigl( \alpha_{n} f\bigl(J_{\lambda}(x_{n})\bigr) \oplus(1- \alpha _{n})T^{n}\bigl(\beta_{n} J_{\lambda}(x_{n}) \oplus(1- \beta_{n})J_{\lambda}(x) \bigr), \\ &\qquad\alpha_{n} f\bigl(J_{\lambda}(x_{n})\bigr) \oplus(1- \alpha_{n})T^{n}\bigl(\beta_{n} J_{\lambda}(x_{n}) \oplus(1- \beta_{n})J_{\lambda}(y) \bigr)\bigr) \\ & \le(1-\alpha_{n}) d\bigl(T^{n}\bigl(\beta_{n} J_{\lambda}(x_{n}) \oplus(1- \beta _{n})J_{\lambda}(x) \bigr), T^{n}\bigl(\beta_{n} J_{\lambda}(x_{n}) \oplus(1- \beta _{n})J_{\lambda}(y)\bigr)\bigr) \\ & \le(1-\alpha_{n}) k_{n} d\bigl(\beta_{n} J_{\lambda}(x_{n}) \oplus(1- \beta _{n})J_{\lambda}(x), \beta_{n} J_{\lambda}(x_{n}) \oplus(1- \beta _{n})J_{\lambda}(y)\bigr) \\ & \le(1-\alpha_{n}) k_{n} (1- \beta_{n}) d \bigl(J_{\lambda}(x), J_{\lambda}(y)\bigr) \\ & \le(1-\alpha_{n}) k_{n} (1- \beta_{n}) d(x, y) \\ &\le(1-\alpha_{n}) k_{n} d(x, y). \end{aligned} $$

By condition (iii), for any given $0 < \epsilon< 1-\gamma$ there exists $n_{0} \ge1$ such that for any $n \ge n_{0}$ we have $k_{n} - 1 < \alpha_{n} \epsilon< \alpha_{n} (1- \gamma) \le\alpha_{n} (k_{n} - \gamma)$, i.e., $(1 -\alpha_{n})k_{n} < 1 - \alpha_{n} \gamma< 1$. Therefore for any $n \ge n_{0}$, $T_{n} : X \to X$ is a contractive mapping. By the Banach contraction principle, there exists a unique fixed point $x_{n+1} \in X$ of $T_{n}$ for each $n \ge n_{0}$. Without loss of generality, in the sequel, we can assume that the following is true for all $n \ge1$:

3.3 $$ \left \{ \textstyle\begin{array}{l} k_{n} - 1 < \alpha_{n} \epsilon,\\ \frac{k_{n} - 1}{\alpha_{n}} < 1- \gamma,\\ (1 -\alpha_{n})k_{n} < 1 - \alpha_{n} \gamma< 1, \end{array}\displaystyle \right . \quad\forall n \ge1. $$

Therefore $\{x_{n}\}$ is well defined.

(II) *Next we prove that* $\{x_{n}\}$ *is bounded.*

In fact, for each $p \in\Gamma: = \operatorname{Fix}(t) \cap A^{-1}(0)$ we have

$$\begin{aligned} d(x_{n+1}, p) & = d\bigl(\alpha_{n} f(y_{n}) \oplus(1- \alpha_{n})T^{n}\bigl( \beta_{n} y_{n} \oplus(1- \beta_{n})y_{n+1} \bigr), p\bigr) \\ & \le\alpha_{n} d\bigl(f(y_{n}), p\bigr) +(1- \alpha_{n}) d\bigl(T^{n}\bigl(\beta_{n} y_{n} \oplus(1- \beta_{n})y_{n+1}\bigr), p\bigr) \\ & \le\alpha_{n} \bigl\{ d\bigl(f\bigl(J_{\lambda}(x_{n}) \bigr), f(p)\bigr) + d\bigl(f(p), p\bigr)\bigr\} \\ & \quad{} + (1- \alpha_{n})k_{n} d\bigl(\beta_{n} y_{n} \oplus(1- \beta_{n})y_{n+1}, p\bigr) \\ & \le\alpha_{n} \gamma d\bigl(J_{\lambda}(x_{n}), p\bigr) + \alpha_{n} d\bigl(f(p), p\bigr) \\ & \quad{} + (1- \alpha_{n})k_{n} d\bigl(\beta_{n} y_{n} \oplus(1- \beta_{n})y_{n+1}, p\bigr) \\ & \le\alpha_{n} \gamma d(x_{n}, p) + \alpha_{n} d \bigl(f(p), p\bigr) \\ & \quad{} + (1- \alpha_{n})k_{n} \bigl\{ \beta_{n} d(x_{n}, p) + (1- \beta_{n}) d(x_{n+1}, p)\bigr\} . \end{aligned} $$

After simplifying, and by using (3.3) we have

$$\begin{aligned} d(x_{n+1}, p) & \le\frac{\alpha_{n} \gamma+ k_{n} \beta_{n} - \alpha_{n} k_{n} \beta_{n}}{1-(1-\alpha_{n} - \beta_{n} +\alpha_{n}\beta_{n})k_{n}} d(x_{n}, p) \\ & \quad{} + \frac{\alpha_{n}}{1-(1-\alpha_{n} - \beta_{n} +\alpha_{n}\beta _{n})k_{n}} d\bigl(f(p), p\bigr) \\ & = \biggl(1 + \frac{(k_{n} -1) - \alpha_{n} k_{n} + \alpha_{n} \gamma}{1-(1-\alpha_{n} - \beta_{n} +\alpha_{n}\beta_{n})k_{n}}\biggr) d(x_{n}, p) \\ & \quad{} + \frac{\alpha_{n}}{1-(1-\alpha_{n} - \beta_{n} +\alpha_{n}\beta _{n})k_{n}} d\bigl(f(p), p\bigr) \\ & \le\biggl(1 + \frac{(\alpha_{n} \epsilon- \alpha_{n} k_{n} + \alpha_{n} \gamma )}{1-(1-\alpha_{n} - \beta_{n} +\alpha_{n}\beta_{n})k_{n}}\biggr) d(x_{n}, p) \\ & \quad{} + \frac{\alpha_{n}}{1-(1-\alpha_{n} - \beta_{n} +\alpha_{n}\beta _{n})k_{n}} d\bigl(f(p), p\bigr) \\ & = \biggl(1 - \frac{k_{n} - \epsilon- \gamma}{1-(1-\alpha_{n} - \beta_{n} +\alpha _{n}\beta_{n})k_{n}}\biggr)\alpha_{n} d(x_{n}, p) \\ & \quad{} + \frac{\alpha_{n}}{1-(1-\alpha_{n} - \beta_{n} +\alpha_{n}\beta _{n})k_{n}} d\bigl(f(p), p\bigr) \\ & \le\biggl\{ 1- \frac{(1-\epsilon- \gamma)\alpha_{n}}{\alpha_{n} + \beta_{n} - \alpha_{n} \beta_{n}}\biggr\} d(x_{n}, p) \\ & \quad{} + \frac{(1 - \gamma- \epsilon)\alpha_{n}}{(1 - \gamma- \epsilon)(\alpha_{n} + \beta_{n} - \alpha_{n} \beta_{n}) } d\bigl(f(p), p\bigr) \\ & \le \max \biggl\{ d(x_{n}, p), \frac{d(f(p), p)}{1 - \gamma- \epsilon}\biggr\} . \end{aligned} $$

By induction we can prove that

$$d(x_{n}, p) \le \max\biggl\{ d(x_{1}, p), \frac{d(f(p), p)}{1 - \gamma- \epsilon} \biggr\} . $$

This implies that the sequence $\{x_{n}\}$ is bounded, so $\{y_{n}\}$, $\{ f(y_{n})\}$ and $\{T^{n}(\beta_{n} y_{n} \oplus (1- \beta_{n})y_{n+1})\}$ are also bounded.

(III) *Next we define a sequence* $\{w_{n}\}$ *by*

3.4 $$ w_{n} = \alpha_{n} f(J_{\lambda}w_{n}) \oplus(1- \alpha_{n})T^{n}(J_{\lambda}w_{n}), \quad\forall n \ge1. $$

By a similar method to the proof of Sect. 1, we can also prove that the sequence $\{w_{n}\}$ is well defined and bounded.

Now we prove that

3.5 $$ \lim_{n \to\infty} d(x_{n+1}, w_{n}) =0. $$

In fact, it follows from (3.1) and (3.4) that

$$\begin{aligned} d(x_{n+1}, w_{n}) &= d\bigl( \alpha_{n} f\bigl(J_{\lambda}(x_{n})\bigr) \oplus(1- \alpha _{n})T^{n}\bigl(\beta_{n} J_{\lambda}(x_{n}) \oplus(1- \beta_{n})J_{\lambda }(x_{n+1}) \bigr), \\ & \quad{}\alpha_{n} f(J_{\lambda}w_{n}) \oplus(1- \alpha_{n})T^{n}(J_{\lambda}w_{n})\bigr) \\ & \le\alpha_{n} d\bigl(f\bigl(J_{\lambda}(x_{n}) \bigr),f(J_{\lambda}w_{n})\bigr)\\ &\quad{} + (1- \alpha_{n}) d \bigl(T^{n}\bigl(\beta_{n} J_{\lambda}(x_{n}) \oplus(1- \beta_{n})J_{\lambda }(x_{n+1})\bigr), T^{n}(J_{\lambda}w_{n})\bigr) \\ & \le\alpha_{n} \gamma d(x_{n}, w_{n}) \\ &\quad{} + (1- \alpha_{n}) k_{n} \bigl\{ \beta_{n} d \bigl(J_{\lambda}(x_{n}), J_{\lambda}w_{n}\bigr)+ (1- \beta_{n}) d\bigl(J_{\lambda }(x_{n+1}), J_{\lambda}w_{n}\bigr)\bigr\} \\ & \le\alpha_{n} \gamma d(x_{n}, w_{n}) + (1- \alpha_{n}) k_{n} \bigl\{ \beta_{n} d(x_{n}, w_{n}) + (1- \beta_{n}) d(x_{n+1}, w_{n})\bigr\} . \end{aligned} $$

After simplifying, and using (3.3), we have

3.6 $$ \begin{aligned}[b] d(x_{n+1}, w_{n}) & \le \frac{\alpha_{n} \gamma+\beta_{n} k_{n} - \alpha_{n} \beta_{n} k_{n}}{1 - (1 - \alpha_{n} - \beta_{n} + \alpha_{n} \beta_{n})k_{n}} d(x_{n}, w_{n}) \\ &= \biggl\{ 1 - \frac{-(k_{n} -1 -\alpha_{n} k_{n} + \alpha_{n} \gamma)}{1 - (1 - \alpha_{n} - \beta_{n} + \alpha_{n} \beta_{n})k_{n}}\biggr\} d(x_{n}, w_{n}) \\ &\le\biggl\{ 1 - \frac{-(\alpha_{n} \epsilon-\alpha_{n} k_{n} + \alpha_{n} \gamma )}{1 - (1 - \alpha_{n} - \beta_{n} + \alpha_{n} \beta_{n})k_{n}}\biggr\} d(x_{n}, w_{n}) \\ & = \biggl\{ 1 - \frac{(k_{n} - \gamma-\epsilon)\alpha_{n}}{1 - (1 - \alpha_{n} - \beta_{n} + \alpha_{n} \beta_{n})k_{n}}\biggr\} d(x_{n}, w_{n}) \\ & \le\biggl(1 - \frac{(1 - \gamma-\epsilon)\alpha_{n}}{\alpha_{n} + \beta_{n} - \alpha_{n} \beta_{n}}\biggr)d(x_{n}, w_{n}) \\ & \le\bigl(1 - (1 - \gamma-\epsilon)\alpha_{n}\bigr) \bigl[d(x_{n}, w_{n-1}) + d( w_{n-1}, w_{n}) \bigr]. \end{aligned} $$

In order to use Lemma 2.9, it should be proved that

3.7 $$ \limsup_{n \to\infty} \frac{d(w_{n-1}, w_{n})}{(1 - \gamma-\epsilon )\alpha_{n}} = 0. $$

Indeed, it follows from Lemma 2.1 that

$$\begin{aligned} d(w_{n}, w_{n -1}) & = d\bigl( \alpha_{n} f(J_{\lambda}w_{n}) \oplus(1- \alpha _{n})T^{n}(J_{\lambda}w_{n}), \\ & \quad{} \alpha_{n-1} f(J_{\lambda}w_{n-1}) \oplus(1- \alpha _{n-1})T^{{n-1}}(J_{\lambda}w_{n-1})\bigr) \\ & \le d\bigl(\alpha_{n} f(J_{\lambda}w_{n}) \oplus(1- \alpha_{n})T^{n}(J_{\lambda}w_{n}), \alpha_{n} f(J_{\lambda}w_{n}) \oplus(1- \alpha_{n})T^{n}(J_{\lambda}w_{n-1})\bigr) \\ & \quad{} + d\bigl( \alpha_{n} f(J_{\lambda}w_{n}) \oplus(1- \alpha _{n})T^{n}(J_{\lambda}w_{n-1}), \alpha_{n} f(J_{\lambda}w_{n-1}) \oplus(1- \alpha_{n})T^{n}(J_{\lambda}w_{n-1}) \bigr) \\ & \quad{} + d\bigl(\alpha_{n} f(J_{\lambda}w_{n-1}) \oplus(1- \alpha _{n})T^{n}(J_{\lambda}w_{n-1}), \\ & \quad{} \alpha_{n-1} f(J_{\lambda}w_{n-1}) \oplus(1- \alpha _{n-1})T^{{n-1}}(J_{\lambda}w_{n-1})\bigr) \\ &\le (1- \alpha_{n-1})d\bigl(T^{n}(J_{\lambda}w_{n}), T^{{n}}(J_{\lambda}w_{n-1})\bigr) \\ & \quad{} + \alpha_{n} d\bigl( f(J_{\lambda}w_{n}), f(J_{\lambda}w_{n-1})\bigr) + \vert \alpha_{n} - \alpha_{n-1} \vert d\bigl(f(J_{\lambda}w_{n-1}), T^{n}(J_{\lambda}w_{n-1})\bigr) \\ &\le(1- \alpha_{n-1})k_{n} d( w_{n}, w_{n-1}) + \alpha_{n} \gamma d( w_{n}, w_{n-1}) + \vert \alpha_{n} - \alpha_{n-1} \vert M^{*}, \end{aligned}$$

where $M^{*} = \sup_{n \ge1} d(f(J_{\lambda}w_{n-1}), T^{n}(J_{\lambda}w_{n-1}))$. After simplifying and using (3.3) we have

$$\begin{aligned} d(w_{n}, w_{n -1}) & \le \frac{1}{-(k_{n} -1 - \alpha_{n} k_{n} + \alpha_{n} \gamma)} \vert \alpha_{n} - \alpha_{n-1} \vert M^{*} \\ & \le\frac{1}{-(\epsilon- k_{n} + \gamma)\alpha_{n}} \vert \alpha_{n} - \alpha _{n-1} \vert M^{*} \\ & \le\frac{1}{(1 - \epsilon- \gamma)\alpha_{n}} \vert \alpha_{n} - \alpha _{n-1} \vert M^{*}. \end{aligned} $$

By the condition (iv) we have

$$\limsup_{n \to\infty} \frac{d(w_{n}, w_{n -1})}{(1 - \epsilon- \gamma )\alpha_{n}} \le\limsup _{n \to\infty} \frac{ \vert \alpha_{n} - \alpha _{n-1} \vert }{(1 - \epsilon- \gamma)^{2}\alpha_{n}^{2}} M^{*} = 0. $$

This implies that (3.7) is true. By Lemma 2.9 and (3.6), we get

3.8 $$ \lim_{n \to\infty} d(x_{n+1}, w_{n}) = 0. $$

(IV) *Next we prove that* $\{x_{n}\}$ *converges strongly to some point* $x^{*} \in\Gamma: = \operatorname{Fix}(T)\cap A^{-1}(0)$ *which is also the unique solution of the following variational inequality:*

3.9 $$ \bigl\langle \overrightarrow{x^{*} f\bigl(x^{*}\bigr)}, \overrightarrow{qx^{*}}\bigr\rangle \ge 0,\quad \forall q \in\Gamma. $$

By (3.8), in order to prove $\{x_{n}\}$ converges strongly to some point $x^{*} \in\Gamma$, it suffices to prove that $\{w_{n}\}$ converges strongly to this point $x^{*} \in\Gamma$.

In fact, it follows from (3.1) and (3.4) that

3.10 $$ \begin{aligned}[b] d\bigl(w_{n}, T^{n} J_{\lambda}(w_{n})\bigr)& = d\bigl(\alpha_{n} f(J_{\lambda}w_{n}) \oplus(1- \alpha_{n})T^{n}(J_{\lambda}w_{n}),T^{n} \bigl(J_{\lambda}(w_{n})\bigr)\bigr) \\ & \le\alpha_{n} d\bigl( f(J_{\lambda}w_{n}), T^{n} \bigl(J_{\lambda}(w_{n})\bigr)\bigr) \to0. \end{aligned} $$

Also for each $p \in\Gamma$, it follows from (2.4) that

3.11 $$ \begin{aligned}[b] d^{2}(w_{n}, p) & = d^{2} \bigl(\alpha_{n} f(J_{\lambda}w_{n}) \oplus(1- \alpha _{n})T^{n}(J_{\lambda}w_{n}), p\bigr) \\ & \le\alpha_{n} d^{2}\bigl(f(J_{\lambda}w_{n}), p\bigr) + (1-\alpha_{n}) d^{2} \bigl(T^{n}(J_{\lambda}w_{n}), p\bigr) \\ & \le\alpha_{n} d^{2}\bigl(f(J_{\lambda}w_{n}), p\bigr) + (1-\alpha_{n})k_{n}^{2} d^{2}\bigl(J_{\lambda}(w_{n}), p\bigr). \end{aligned} $$

After simplifying, we have

3.12 $$ - d^{2}\bigl(J_{\lambda}(w_{n}), p\bigr)\le\frac{1}{(1-\alpha_{n})k_{n}^{2}} \bigl\{ \alpha_{n} d^{2}\bigl(f(J_{\lambda}w_{n}), p\bigr) - d^{2}( w_{n}, p)\bigr\} . $$

Again since $J_{\lambda}$ is firmly nonexpansive, we have

$$\begin{aligned} d^{2}\bigl(J_{\lambda}(w_{n}), p\bigr) &\le\bigl\langle \overrightarrow{J_{\lambda}(w_{n})p} , \overrightarrow{w_{n} p} \bigr\rangle \\ & = \frac{1}{2} \bigl\{ d^{2}\bigl(J_{\lambda}(w_{n}), p\bigr) + d^{2}(p, w_{n}) - d^{2} \bigl(J_{\lambda}(w_{n}), w_{n}\bigr)\bigr\} . \end{aligned} $$

This together with (3.12) shows that

3.13 $$ \begin{aligned}[b] d^{2}\bigl(J_{\lambda}(w_{n}), w_{n}\bigr) & \le d^{2}(p, w_{n}) - d^{2} \bigl(J_{\lambda}(w_{n}), p\bigr) \\ & \le d^{2}(p, w_{n}) + \frac{1}{(1-\alpha_{n})k_{n}^{2}} \bigl\{ \alpha_{n} d^{2}\bigl(f(J_{\lambda}w_{n}), p \bigr) - d^{2}(w_{n}, p)\bigr\} \to0. \end{aligned} $$

From (3.10) and (3.13) one gets

3.14 $$ \lim_{n \to\infty} d\bigl(J_{\lambda}(w_{n}), T^{n} J_{\lambda}(w_{n})\bigr) = 0. $$

By the assumption that *T* is uniformly asymptotic regularity, from (3.14) we obtain

3.15 $$ \begin{aligned}[b] d\bigl(J_{\lambda}(w_{n}), TJ_{\lambda}(w_{n})\bigr)& \le d\bigl(J_{\lambda}(w_{n}), T^{n} J_{\lambda}(w_{n})\bigr) + d\bigl(T^{n} J_{\lambda}(w_{n}), T^{n+1} J_{\lambda}(w_{n}) \bigr) \\ & \quad{} + d\bigl(T^{n+1} J_{\lambda}(w_{n}), TJ_{\lambda}(w_{n})\bigr) \\ & \le(1 + k_{1})d\bigl(J_{\lambda}(w_{n}), T^{n} J_{\lambda}(w_{n})\bigr) + d\bigl(T^{n} J_{\lambda}(w_{n}), T^{n+1} J_{\lambda}(w_{n})\bigr) \\&\to0 \quad(\text{as } n \to\infty). \end{aligned} $$

Since $\{w_{n}\}$ is bounded, by Lemma 2.2(1) there exists a subsequence $\{w_{n_{i}}\}$ of $\{w_{n}\}$ which Δ-converges to some point $x^{*}$. It then follows from (3.13) that there exists a subsequence $\{J_{\lambda}(w_{n_{i}})\}$ of $\{J_{\lambda}(w_{n})\}$ which Δ-converges to $x^{*}$. Thus, from (3.13), (3.15), and Lemma 2.6(2), we obtain $x^{*} \in \operatorname{Fix}(T)\cap A^{-1}(0) =\Gamma$.

Next we prove that $\lim_{n \to\infty} w_{n} = x^{*}$ which is also the unique solution of the variational inequality (3.9).

In fact, it follows from Lemma 2.1(4) that

$$\begin{aligned} d^{2}\bigl(w_{n}, x^{*}\bigr) & = d^{2}\bigl(\alpha_{n} f(J_{\lambda}w_{n}) \oplus(1- \alpha _{n})T^{n}(J_{\lambda}w_{n}), x^{*}\bigr) \\ &\le\alpha_{n}^{2} d^{2}\bigl(f(J_{\lambda}w_{n}), x^{*}\bigr) + (1-\alpha_{n})^{2} d^{2}\bigl(T^{n}(J_{\lambda}w_{n}), x^{*}\bigr) \\ & \quad{} + 2\alpha_{n} (1-\alpha_{n}) \bigl\langle \overrightarrow{f(J_{\lambda}w_{n}) x^{*}}, \overrightarrow{T^{n}(J_{\lambda}w_{n}) x^{*}} \bigr\rangle \\ &\le\alpha_{n}^{2} d^{2}\bigl(f(J_{\lambda}w_{n}), x^{*}\bigr) + (1-\alpha_{n})^{2} k_{n}^{2} d^{2}\bigl(J_{\lambda}w_{n}, x^{*}\bigr) \\ & \quad{} + 2\alpha_{n} (1-\alpha_{n}) \bigl\{ \bigl\langle \overrightarrow {f(J_{\lambda}w_{n}) x^{*}}, \overrightarrow{T^{n} \bigl(J_{\lambda}(w_{n})\bigr)J_{\lambda}(w_{n})} \bigr\rangle \\ & \quad{} + \bigl\langle \overrightarrow{ f(J_{\lambda}w_{n}) f \bigl(x^{*}\bigr)}, \overrightarrow{J_{\lambda}(w_{n})x^{*}} \bigr\rangle + \bigl\langle \overrightarrow {f\bigl(x^{*}\bigr) x^{*}}, \overrightarrow{J_{\lambda}(w_{n})x^{*}} \bigr\rangle \bigr\} \\ &\le\alpha_{n}^{2} d^{2}\bigl(f(J_{\lambda}w_{n}), x^{*}\bigr) + (1-\alpha_{n})^{2} k_{n}^{2} d^{2}\bigl(w_{n}, x^{*}\bigr) \\ & \quad{} + 2\alpha_{n} (1-\alpha_{n})\bigl\{ d \bigl(f(J_{\lambda}w_{n}), x^{*}\bigr) d\bigl(T^{n} \bigl(J_{\lambda}(w_{n})\bigr), J_{\lambda}(w_{n}) \bigr) \\ & \quad{} + \gamma d^{2}\bigl(w_{n}, x^{*}\bigr) + \bigl\langle \overrightarrow{f\bigl(x^{*}\bigr) x^{*}}, \overrightarrow{J_{\lambda}(w_{n})x^{*}} \bigr\rangle \bigr\} . \end{aligned}$$

After simplifying, we have

3.16 $$ \begin{aligned}[b] d^{2}\bigl(w_{n}, x^{*}\bigr) & \le\frac{\alpha_{n}}{1 - 2\alpha_{n}(1-\alpha_{n})\gamma- (1- \alpha_{n})^{2} k_{n}^{2}} \bigl\{ \alpha_{n} d^{2} \bigl(f(J_{\lambda}w_{n}), x^{*}\bigr) \\ & \quad{} + 2(1-\alpha_{n}) \bigl[ d\bigl(f(J_{\lambda}w_{n}), x^{*}\bigr) d\bigl(T^{n}\bigl(J_{\lambda}(w_{n})\bigr), J_{\lambda}(w_{n})\bigr) \\ & \quad{} + \bigl\langle \overrightarrow{f\bigl(x^{*}\bigr) x^{*}}, \overrightarrow {J_{\lambda}(w_{n})x^{*}} \bigr\rangle \bigr]\bigr\} . \end{aligned} $$

We use

3.17 $$ \begin{aligned}[b] & \frac{\alpha_{n}}{1 - 2\alpha_{n}(1-\alpha_{n})\gamma- (1- \alpha_{n})^{2} k_{n}^{2}} \\ &\quad= \frac{1}{(2-\alpha_{n})k_{n}^{2} - 2(1-\alpha_{n})\gamma+ \frac{(1 - k_{n})(1 + k_{n})}{\alpha_{n}}} \to\frac{1}{2(1-\gamma)} \quad(\text{as } n \to\infty). \end{aligned} $$

Again since $\{J_{\lambda}(w_{n_{i}})\}$ Δ-converges to $x^{*} \in \Gamma$, by Lemma 2.2(2), we have

3.18 $$ \lim_{n_{i} \to\infty}\bigl\langle \overrightarrow{f\bigl(x^{*}\bigr) x^{*}}, \overrightarrow{J_{\lambda}(w_{n_{i}})x^{*}} \bigr\rangle = \limsup _{n_{i} \to \infty}\bigl\langle \overrightarrow{f\bigl(x^{*}\bigr) x^{*}}, \overrightarrow{J_{\lambda}(w_{n_{i}})x^{*}} \bigr\rangle \le0. $$

It follows from (3.14), (3.16), (3.17) and (3.18) that

3.19 $$ \lim_{n_{i} \to\infty} d\bigl(w_{n_{i}}, x^{*}\bigr) =0. $$

Next we prove that $x^{*}$ is a solution of variational inequality (3.9). In fact, for any $q \in\Gamma$, it follows from Lemma 2.1(4) that (for the sake of convenience we denote $\{w_{n_{i}}\}$ by $\{w_{i}\}$)

$$\begin{aligned} d^{2}(w_{i},q) & = d^{2}\bigl(\alpha_{n} f(J_{\lambda}w_{i}) \oplus(1- \alpha _{i})T^{i}(J_{\lambda}w_{i}), q\bigr) \\ & \le\alpha_{i} d^{2}\bigl( f(J_{\lambda}w_{i}), q\bigr) + (1- \alpha_{i}) d^{2}\bigl( T^{i}(J_{\lambda}w_{i}), q\bigr) \\ & \quad{} -\alpha_{i} (1- \alpha_{i}) d^{2} \bigl(f(J_{\lambda}w_{i}),T^{i}(J_{\lambda}w_{i})\bigr) \\ & \le\alpha_{i} d^{2}\bigl( f(J_{\lambda}w_{i}), q\bigr) + (1- \alpha_{i})k_{i}^{2} d^{2}( w_{i}, q) \\ & \quad{} -\alpha_{i} (1- \alpha_{i}) d^{2} \bigl(f(J_{\lambda}w_{i}),T^{i}(J_{\lambda}w_{i})\bigr). \end{aligned} $$

After simplifying we have

3.20 $$ d^{2}(w_{i},q) \le\frac{1}{\frac{1-k_{i}^{2}}{\alpha_{i}} + k_{i}^{2}}\bigl\{ d^{2} \bigl( f(J_{\lambda}w_{i}), q\bigr) - (1- \alpha_{i}) d^{2}\bigl(f(J_{\lambda}w_{i}),T^{i}(J_{\lambda}w_{i})\bigr)\bigr\} . $$

On the other hand, it follows from (3.19) and (3.13) that $w_{i} \to x^{*}$ and $J_{\lambda}(w_{i}) \to x^{*}$ (as $i \to\infty$). Hence $f(J_{\lambda}(w_{i})) \to f(x^{*})$. Again by (3.14) and condition (iii), $T^{i}(J_{\lambda}w_{i}) \to x^{*}$ and $\frac{1}{\frac{1-k_{i}^{2}}{\alpha_{i}} + k_{i}^{2}} \to1$ (as $i \to\infty$). Letting $i \to\infty$ in (3.20) we have

$$d^{2}\bigl(x^{*},q\bigr) \le d^{2}\bigl(f\bigl(x^{*}\bigr), q \bigr) - d^{2}\bigl(f\bigl(x^{*}\bigr), x^{*}\bigr), $$

i.e.,

$$0 \le d^{2}\bigl(f\bigl(x^{*}\bigr), q\bigr) - d^{2}\bigl(f \bigl(x^{*}\bigr), x^{*}\bigr) - d^{2}\bigl(x^{*},q\bigr). $$

Hence we have

$$\bigl\langle \overrightarrow{x^{*} f\bigl(x^{*}\bigr)}, \overrightarrow{q x^{*}}\bigr\rangle = \frac{1}{2}\bigl\{ d^{2}\bigl(f\bigl(x^{*}\bigr), q\bigr) - d^{2}\bigl(f\bigl(x^{*}\bigr), x^{*}\bigr) - d^{2}\bigl(x^{*},q \bigr)\bigr\} \ge0,\quad \forall q \in\Gamma, $$

i.e., $x^{*}$ is a solution of variational inequality (3.9). If there exists another subsequence $\{w_{n_{k}}\}$ of $\{w_{n}\}$ which Δ-converges to $y^{*}$. By the same argument, we know that $y^{*} \in\Gamma$ which solves the variational inequality (3.9). Therefore we have

$$\begin{gathered} \bigl\langle \overrightarrow{x^{*} f\bigl(x^{*}\bigr)}, \overrightarrow{y^{*} x^{*}}\bigr\rangle \ge0, \\ \bigl\langle \overrightarrow{y^{*} f\bigl(y^{*}\bigr)}, \overrightarrow{x^{*} y^{*}} \bigr\rangle \ge0. \end{gathered} $$

Adding the above two inequalities, we obtain

$$\begin{aligned} 0 & \le \bigl\langle \overrightarrow{x^{*} f\bigl(x^{*} \bigr)}, \overrightarrow{y^{*} x^{*}} \bigr\rangle - \bigl\langle \overrightarrow{y^{*} f \bigl(y^{*}\bigr)}, \overrightarrow{ y^{*}x^{*}}\bigr\rangle \\ & = \bigl\langle \overrightarrow{x^{*} f\bigl(y^{*}\bigr)}, \overrightarrow{y^{*} x^{*}} \bigr\rangle + \bigl\langle \overrightarrow{f\bigl(y^{*}\bigr)f\bigl(x^{*}\bigr)}, \overrightarrow{y^{*} x^{*}}\bigr\rangle \\ & \quad{} - \bigl\langle \overrightarrow{y^{*} x^{*}}, \overrightarrow{y^{*} x^{*}} \bigr\rangle - \bigl\langle \overrightarrow{x^{*} f\bigl(y^{*}\bigr)}, \overrightarrow{y^{*} x^{*}} \bigr\rangle \\ & \le\gamma d^{2}\bigl(y^{*}, x^{*}\bigr) - d^{2}\bigl(y^{*}, x^{*} \bigr) < 0. \end{aligned} $$

This is a contradiction, and so $x^{*} = y^{*}$. Hence $\{w_{n}\}$ converges strongly to $x^{*}$. By (3.8) one shows that $\{x_{n}\}$ converges strongly to $x^{*}$.

This completes the proof of Theorem 3.1. □
